# Advancements in genomic crop techniques and considerations for regulation and food safety

**DOI:** 10.1007/s11248-025-00467-4

**Published:** 2025-11-27

**Authors:** Gijs W. Spaans, Jan Pieter van der Berg, Lianne M. S. Bouwman, Gijs A. Kleter

**Affiliations:** Wageningen Food Safety Research (WFSR), Wageningen University & Research, Akkermaalsbos 2, 6708 WB Wageningen, The Netherlands

**Keywords:** Genome editing, New genomic techniques, CRISPR-Cas, TALENs, TILLING, Transposons

## Abstract

Advancements in genomic crop techniques have led to the development of new genetic technologies, such as base- and prime editing, but improvements have been made to existing conventional techniques as well. Fields in which these advancements occur include targeted mutagenesis, conventional random mutagenesis, and developments with null segregants, *e.g.*, crops from which transgenic elements have been crossed out. In this review, we describe the developments in these three fields and provide considerations concerning regulatory and safety aspects. Because of differences in legislation of modern biotechnology between countries or regions, regulatory challenges are to be expected given the ongoing developments in genomic crop techniques. Moreover, the nature of the mutations induced with these newly developed techniques is not different from those induced with conventional techniques, making the modified crop plants indistinguishable from non-modified counterparts of the same crop species. Thus, enforcement of regulations cannot solely rely on technical analytical methods. Also, potential off-target or unintended effects in the primary mutants remain underexplored. Yet, these do not raise safety concerns owing to the experience with the crop breeding practice of iterative cycles for desirable traits selection, as well as the segregation and discard of unwanted phenotypes. Given that regulation will always change after innovation and developments within the sector advance rapidly, we advocate that both authorities and the breeding sector pro-actively implement a food safety culture. Such a safety culture will help developers of genomic technologies in crops to identify potential food safety issues at an early stage of development of future products.

## Introduction

Genomic techniques applied in the crop breeding process undergo continuous development, and these developments can for instance aim at enhancing crops for food and feed. Such genomic enhancements can be initiated through more conventional random mutagenesis techniques as well as newer targeted mutagenesis techniques. Both of these mutagenesis techniques induce heritable changes in the DNA to create a variety in traits between agricultural plants, but the type of mutation induced in the plant as well as its regulatory implications can differ with the applied technique.

For decades, plant breeding has benefitted from so-called random mutation breeding, for which a physical or chemical “mutagen” is used to induce random mutations in the host plant’s genome. In 1928, the first experiments with random mutation breeding were performed. These experiments introduced mutations in barley and maize seeds by means of X-rays (EFSA 2021; Kharkwal 2012). In the 1930s, the first mutant variety developed using X-rays was introduced on the market and since then, other radiation-based mutagenesis techniques such as gamma-rays have emerged (Kharkwal 2012). Generally, X-rays and gamma-rays induce diverse types of mutations, including point mutations, insertions and deletions (InDels), as well as chromosomal rearrangements.

Induced mutagenesis involving the use of mutagenic chemicals became more frequently applied in the 1940s, and, in the period of 1950 to 1970, plant breeders started making widespread use of this technology. Researchers from various nations around the world successfully developed mutant varieties for a variety of crops (Kharkwal 2012). Nowadays, a wide range of chemicals is available for plant mutation breeding, such as alkylating agents (Holme et al. 2019; Oladosu et al. 2016; Shelake et al. 2019; Viana et al. 2019) and sodium azide (Spencer-Lopes et al. 2018; Viana et al. 2019). These chemicals mainly introduce point mutations.

Since the 1990s, plant breeders also started to adopt techniques that make use of exposing materials to high energy irradiation or to a combination of cosmic radiation and microgravity (*i.e.*, space breeding). For these types of radiation, it is known that the damage in the DNA is clustered (Asaithamby and Chen 2011; Jo and Kim 2019; Wang et al. 2022), which is in contrast to conventional forms of random mutagenesis whereby DNA damage is more evenly spread across the genome (Huefner et al. 2014).

In the mid-1990s, the first transgenic, genetically modified (GM) crop plants were commercialized. In these crops, “foreign” transgenes had been introduced by means of recombinant DNA technology. In many cases, these GM plants had been engineered to exhibit properties of agronomic interest, such as insect resistance or herbicide tolerance (ISAAA 2018).

A major development that occurred in the last two decades is the development of New Genomic Techniques (NGTs) that can induce mutations in predefined locations: targeted or site-directed mutagenesis techniques. NGTs include genome editing technologies such as Zinc-Finger Nucleases (ZFNs), Transcription Activator-Like Effector Nucleases (TALENs), and Clustered Regularly Interspaced Short Palindromic Repeats (CRISPR) and associated proteins (CRISPR-Cas). These nuclease complexes are directed to specific DNA sites where they can exert various modes of action, including gene inactivation, activation, interference, insertion, and modulation. Before the discovery of the potential application of CRISPR-Cas9 in gene editing in 2012 (Jinek et al. 2012), ZFNs and TALENs were already employed for this purpose. At the time of this writing, CRISPR-Cas9 from the bacterium *Streptococcus pyogenes* (SpCas9) has become the most widely used nuclease complex because of its robustness with targeting and cleavage of DNA. However, the occurrence of off-target editing by SpCas9 can still be relatively high (Anzalone et al. 2020). Thus, research remains focused on continuous improvement of existing nuclease complexes for genome editing to increase specificity. On the other hand, new genetic technologies are being developed to make it easier to, for example, enable larger genetic alterations, multiplex gene editing, or editing of the organelle genome in plant cells. Currently, various commodity crops developed with the aid of NGTs are already commercially cultivated in Northern America and elsewhere. Notable examples include herbicide-tolerant oilseed rape developed through oligonucleotide-directed mutagenesis, and maize with the waxy starch phenotype developed with CRISPR Cas9 (Cibus 2025; Gao et al. 2020).

Modern biotechnological techniques such as NGTs are able to introduce traits derived from other species into plants, resulting in transgenic crops. When these transgenic elements are selected out in the progeny of the crop, the non-transgenic progeny is called a negative or null segregant. Null segregants can have various origins and have a strong link with various techniques used for the development of genetically modified organisms (GMOs).

**Fig. 1:**
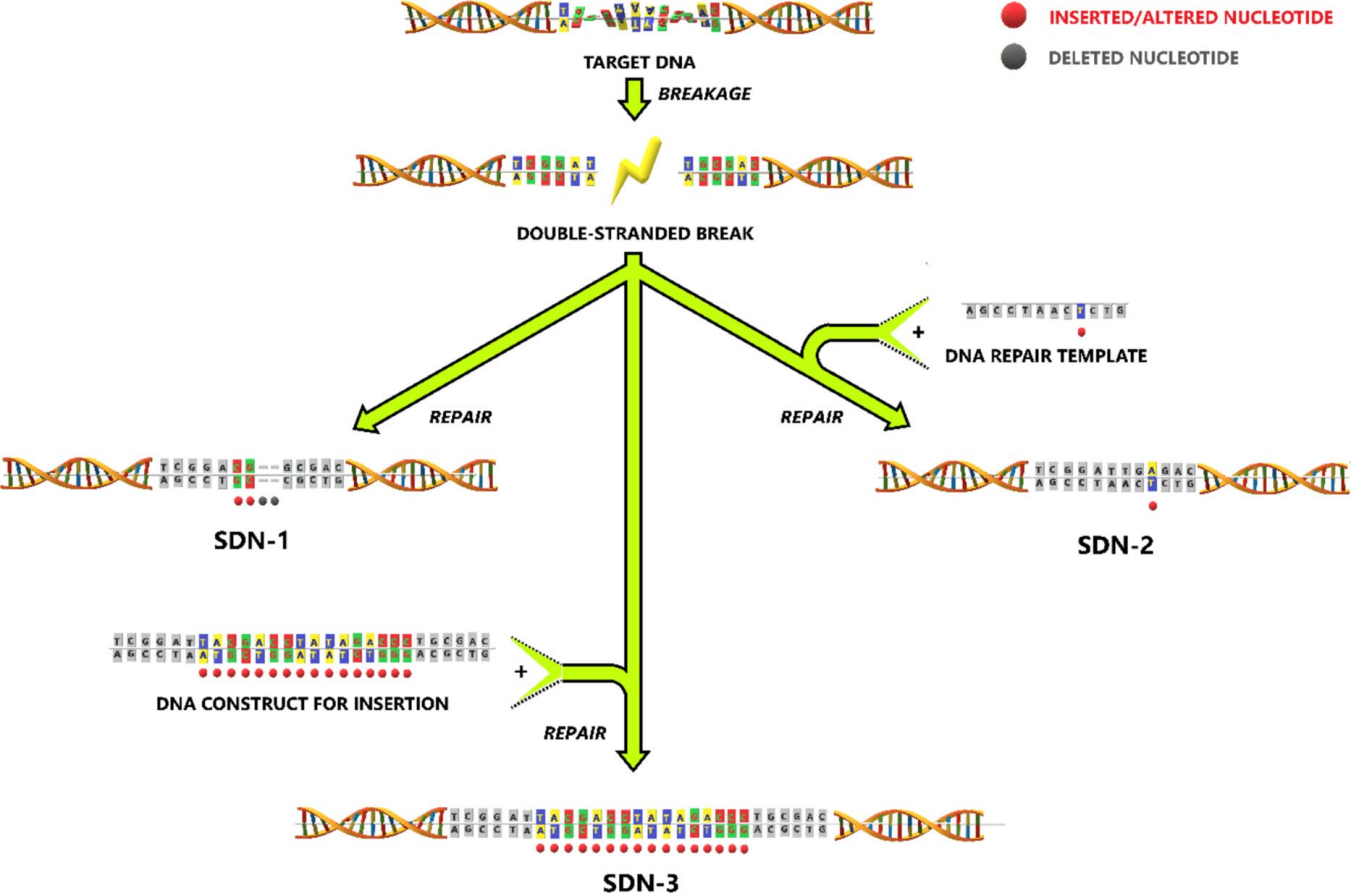
The various DNA edits introduced by site-directed nucleases (SDN) 1, 2, and 3. SDN-1 introduces small random mutations such as deletions, insertions, and substitutions. SDN-2 makes use of a template DNA to cause a targeted mutation. SDN-3 involves the insertion of a large DNA fragment. Red dots indicate introduced or mutated bases, while grey ones indicate deleted bases

The abovementioned advancements in genetic crop improvement techniques are considered differently by regulatory authorities around the world. In many countries, chemical and radiation mutagenesis are exempted from GMO regulations based on their history of safe use (*e.g.*, EU 2001b). On the other hand, the regulatory approach regarding NGTs and null segregants differs significantly between countries. For example, in 2018, the European Court of Justice ruled in case C-528/16 that organisms developed with NGTs are GMOs based on their definition in Directive 2001/18/EC and therefore not excluded from the scope of GMO regulations (CJEU 2018). Application of these so-called site-directed nucleases (SDNs) was classified by the EFSA GMO panel in three categories: SDN-1, SDN-2, and SDN-3 (Fig. 1). When applying SDN-1, small random mutations such as deletions, insertions, and substitutions are introduced in the genome. SDN-2 is defined as making use of template DNA to cause a targeted mutation, while SDN-3 involves the insertion of a large DNA fragment at a specific location in the genome (EFSA GMO Panel 2020). In other countries such as Argentina, Australia, and the USA, these techniques are assessed more on a case-by-case based approach rather than a technique-based approach.

In areas with a process-based approach, GMO regulations may apply to null segregants. In the EU for example, null segregants are considered not to have a history of safe use; consequently, they would not be exempted from GMO regulations. Other countries have explicitly excluded null segregants from their regulations on GMOs, such as Australia, Argentina and Japan (OGTR, 2025; Whelan and Lema 2019; Tsuda et al. 2019), given the absence of transgenic DNA in the end product.

Overall, many advancements involve efforts to improve specificity, and thus safety of certain genetic techniques. However, entirely new genetic technologies are being developed as well, and these new technologies may be associated with unknown off-target or untargeted effects. Thus, progressive insights into these effects need to be taken into account. Also, advancements in genomic techniques may have significant regulatory implications. In this perspectives review, we give examples of advancements in genetic crop improvement techniques and provide considerations for their regulation and safety assessment.

## Examples of developments in genetic crop improvement techniques

### Advancements in gene editing

#### CRISPR-Cas related techniques

In the last decade, a large range of CRISPR-Cas related techniques have been developed. These consist of, among others, newly engineered Cas9 and Cas12 proteins but also CRISPR activation (CRISPRa), CRISPR interference (CRISPRi), CRISPR-combo, base editing, and prime editing systems (Table 1). Besides Cas9 variants, Cas12 variants are often utilized in plant biotechnology as well (Anzalone et al. 2020). Both Cas9 and Cas12 orthologs require a single guide RNA (sgRNA) guiding the protein to the specific target location in the DNA. The advantage of Cas12 over Cas9 is the fact that the sgRNA of Cas12 only requires its CRISPR RNA (crRNA), while the sgRNA of Cas9 requires a fusion of crRNA and synthesized trans-activating crRNA (tracrRNA). This facilitates the use of smaller constructs and thereby simplifies multiplex genome editing (Zetsche et al. 2017). Newly engineered Cas9 or Cas12 proteins focus mainly on reducing the size of viral delivery systems (Gong et al. 2024; Sun et al. 2024), making them compatible with different PAM sites (Gong et al. 2024; He et al. 2024; H. Wang et al. 2024a, b), and diminishing the occurrence of related off-target effects (Deka 2024; Tripathi et al. 2023; Tyumentseva et al. 2023).

**Table 1:** Overview of recent developments on CRISPR-Cas related techniques

Technique	Mechanism	Effect/outcome of the application	Precision
Cas9	Robust targeting/cleavage of DNA	Ranging from small InDels (insertions and deletions) to integration of large stretches of genetic material (e.g., transgenes)	Relatively high number of off-target edits
Cas12	Only crRNA needed	Similar to Cas9 (InDels, larger insertions), also utilized for multiplex genome editing	Fewer off-target edits than most Cas9 variants
CRISPRa	dCas coupled with transcription activator proteins	(Multiplexed) gene activation leading to enhanced expression of target genes	No DSB required
CRISPRi	dCas-sgRNA binds to target obstructing transcription	(Multiplexed) gene silencing leading to repression of gene expression at the transcriptional level	Efficient and low off-target activity in bacteria and eukaryotes
CRISPR combo	Uses Cas9 instead of dCas9 with CRISPRa or CRISPRi	Simultaneous combination of gene activation/silencing and gene/base editing	Equal efficiency as stand-alone techniques
Base editing	nCas9 merged with cytidine/adenine deaminase	Transitions from C-G to T-A base pairs (CBEs) or from A-T to G-C base pairs (ABEs)	Few off-target edits
Prime editing	nCas9 combined with reverse transcriptase	Allows editing for all 12 combination swaps	Low off-target but low efficiency in plants

*CRISPRa* CRISPR activation, *CRISPRi* CRISPR interference, *crRNA* CRISPR RNA, *dCas* dead Cas, *sgRNA* single guide RNA, *nCas* Cas nickase, *DSB* Double Stranded Break

To achieve gene editing activity, the ribonucleoprotein (RNP) complex, consisting of the Cas protein and guide RNA, has to reach the nucleus to be able to make the desired genetic alterations. Cellular delivery and expression of this RNP complex can be achieved using several different methods. A straightforward way to achieve expression of the RNP components is by means of DNA, for example encoded on plasmid DNA or linear DNA constructs which are integrated in the host genome. This option has certain benefits: genes of the CRISPR-Cas components are expressed in a stable manner and constructs can be easily amplified using bacteria. It does, however, mean that transgenic elements are incorporated in the host genome (Lin et al. 2022). Alternative delivery systems concern the use of mRNA, which is translated in the target cell, or the direct delivery of the RNPs. In both of these alternative strategies, the introduction is transient, and persistence of mRNA and RNP depends on their half-life and cellular turnover (Lin et al. 2022).

CRISPRa systems can activate specific genes by means of promoting the potential of specific plant functions instead of deactivating certain functions (for which ‘conventional’ CRISPR-Cas systems are often employed). These CRISPRa systems are RNA-guided CRISPR activation systems that can in general activate one or multiple genes and are based on a de-activated Cas (dCas) protein (also called dead-Cas system) (Maeder et al. 2013; Pan et al. 2021a, b; Perez-Pinera et al. 2013). CRISPRi is also based on a dCas9 protein and causes gene silencing at the DNA level (knock-down). This mechanism is based on blockage of DNA transcription when the dCas-sgRNA complex binds to a target sequence within an open reading frame, thereby obstructing transcription (McCarty et al. 2020). In addition, simultaneous genome editing and genome activation or silencing at different locations in the genome can be achieved with CRISPR-Combo. A CRISPR-Combo system can be designed using Cas9 instead of dCas9 in combination with a CRISPRa or CRISPRi system (Pan et al. 2022).

**Fig. 2:**
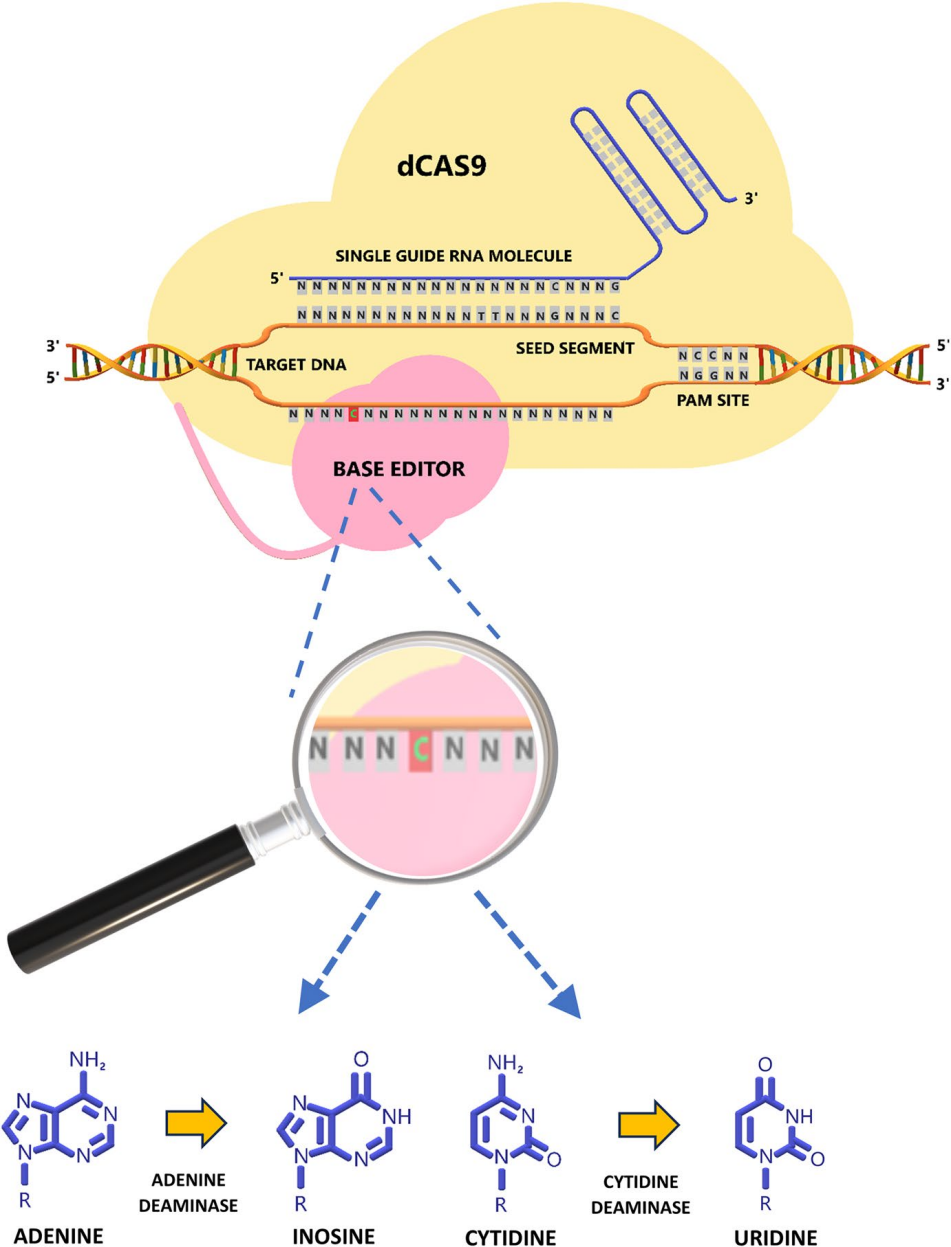
The principle of base editing. A complex of sgRNA bound to a deactivated form of Cas9 (dCas9) is still capable of binding and unwinding target DNA near a PAM recognition site. A base editor enzyme which is covalently linked to dCas9, such as cytidine deaminase or adenine deaminase, deaminates cytidine or adenine to uridine or inosine, respectively, within an editing window in the proximity of the recognition site. The host's repair system will then fill in the corresponding, pairing positions in the opposing strand with the complementary bases of adenine and cytosine, respectively. Following DNA replication, the edited bases cytidine and adenine will ultimately be replaced with thymidine and guanine, respectively

Base editing is a recent development that aims to overcome the inefficient and/or inaccurate repair mechanisms that are involved when genome editing techniques initiate a double-stranded break (DSB). No DSB is introduced with base editing since base editors use nickase or dCas effectors, which make them more precise (Anzalone et al. 2020) (Fig. 2). Different categories of DNA base editors exist, among others, cytosine base editors (CBEs) enabling transitions from C·G to T·A base-pairs and adenine base editors (ABEs) enabling transitions from A·T to G·C base pairs. CBEs and ABEs can cover 4 out of 12 possible base substitutions and only catalyze base transition mutations, meaning substitution of nucleobases within the same purine (A and G) or pyrimidine (C and T) chemical group (C → T, A → G, T → C, G → A) (Molla et al. 2020, 2021).

**Fig. 3:**
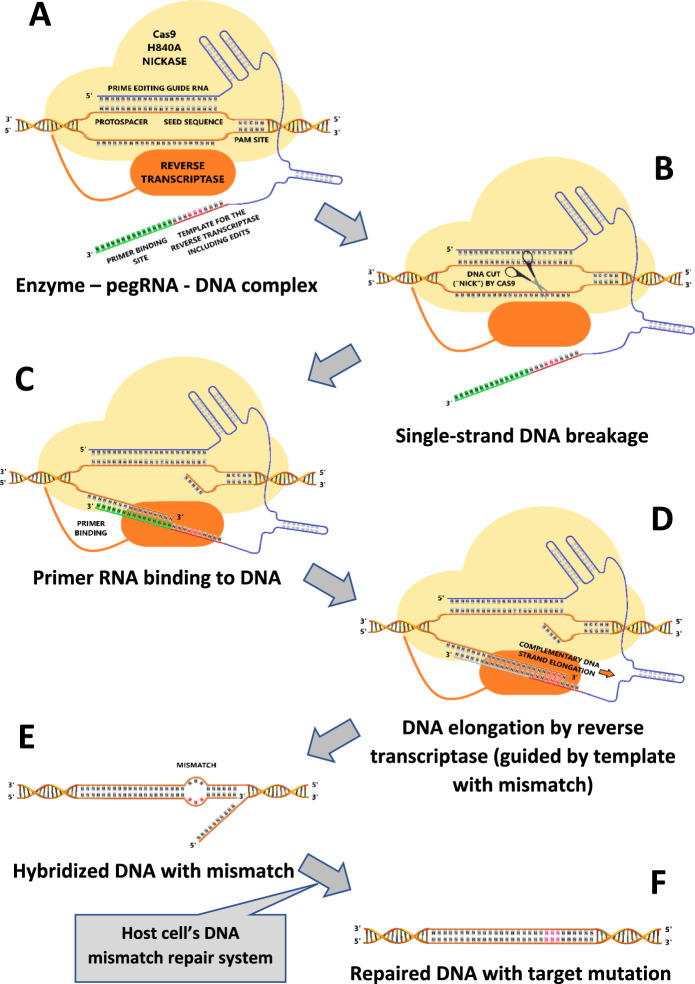
The principle of prime editing. A complex of “prime editing guide RNA” (pegRNA, sgRNA with a RNA template for reverse transcription) bound to a mutant form (“nickase”) of Cas9 introduces a single-strand break (“nick”) in the target DNA near the PAM site. One nicked DNA strand hybridizes with the primer binding site on the pegRNA. The reverse transcriptase covalently linked to the mutant Cas9 will elongate this strand with a sequence complementary to the template, which is identical to the target sequence except for specific edits. After rehybridization and repair by the host DNA repair system, the new edits become fixed in the target DNA

Prime editing, also called ‘search-and-replace’ genome editing, does likewise not require a DSB and enables, in contrast to base editing, the editing of bases for all 12 possible combination swaps. Prime editing was described for the first time in 2019 (Anzalone et al. 2019). A prime editor consists of a Cas9 nickase (nCas9) combined with an engineered reverse transcriptase (RT) (Molla et al. 2020, 2021) (Fig. 3). However, the efficiency of prime editing needs improvement before it can be applied as a useful and successful gene editing tool in plants (Lin et al. 2020; Perroud et al. 2022; Tang et al. 2020; Xu et al. 2020).

#### Mobile genetic elements

Genetic sequences that are able to jump from one part of the DNA to the other are called mobile genetic elements. Of the many mobile genetic elements that exist in nature, four types are used for genetic engineering purposes: DNA transposons, CRISPR-associated transposons, retrotransposons (also called RNA transposons), and retrons (Hwang et al. 2024). These types are classified based on their specificity and their translocation mechanism. DNA transposons and retrotransposons can cause mutations that are more randomly spread over the genome, while CRISPR-associated transposons and retrons are more site-specific. Also, DNA transposons and CRISPR-associated transposons make use of a cut-and-paste translocation mechanism, which means that during transpositioning, these transposons are removed from their original location and translocated to another location in the genome. DNA transposons make use of DNA intermediates when translocating, and CRISPR-associated transposons are DNA transposons that are accompanied by CRISPR RNA (Hwang et al. 2024; Saraswat et al. 2023). On the other hand, retrotransposons and retrons make use of a copy-and-paste translocation mechanism, signifying that these transposons stay in their original position, and only a copy of the transposon is translocated to another location in the genome (Hwang et al. 2024; Saraswat et al. 2023). Retrotransposons make use of RNA intermediates when translocating, and retrons are DNA segments that code for a reverse transcriptase enzyme making multiple replications of single stranded DNA (Hieu and Toan 2023).

Examples of transposon systems that have been studied for potential use in genome editing in the past years are the Ty3/Gypsy and ONSEN retrotransposons (An et al. 2023; Dickinson et al. 2023), the piggyBac DNA transposon (Kishi-Kaboshi et al. 2023; Nishizawa-Yokoi and Toki 2023), and the Pong CRISPR-associated transposon (Liu et al. 2024). The Ty3/Gypsy and ONSEN retrotransposons have been utilized to increase the expression of a specific gene in soybean and *Arabidopsis*, respectively (An et al. 2023; Dickinson et al. 2023). The DNA transposon piggyBac can repair the excision site without leaving a footprint and has been observed to cause efficient, safe, and stable transpositions (Nishizawa-Yokoi and Toki 2023; Saraswat et al. 2023). PiggyBac can translocate cargo DNA larger than 100 kb (Rostovskaya et al. 2012), and as such is able to remove transgenes, such as the Cas9 expression cassette, from plant DNA (Kishi-Kaboshi et al. 2023). Liu et al. (2024) managed to demonstrate the functionality of the CRISPR-associated transposon system with the rice Pong DNA transposon. This transposon was co-expressed with Cas proteins and successfully inserted a gene in *Arabidopsis* and soybean. These transposon systems may prove to be useful in crops where outcrossing of CRISPR-associated genetic material is not feasible, such as in clonally propagated crop plants (Goralogia et al. 2021). As for other transferable vectors, the regulatory safety assessment of GM plants will consider the genetic stability of the modification. For example, when the modification is stable, the vector only enables the mutation of interest to occur, and there is no potential for remobilization of the transposon. All in all, developments on mobile genetic elements focus on efforts to explore or further improve transposon systems, but, to the knowledge of the authors, no such systems have yet been developed for commercial purposes.

#### Transcription activator-like effectors nucleases (TALENs)

At the time of writing, the stable insertion of heritable foreign genes into plant organellar genomes by means of CRISPR-Cas9 has remained unfeasible (Arimura and Nakazato 2024), as no delivery method is able to efficiently deliver the guide RNA needed for the gene editing system (J. Wang et al. 2024a, b). On the other hand, Transcription Activator-Like Effectors Nucleases (TALENs) are able to reach into the mitochondria. Therefore, in the past years, efforts have focused on the development of mitochondrial and plastid (chloroplast) genome editing through TALENs. Concerning mitochondrial genome editing, already a substantial amount of research has been conducted on mitochondria-targeted TALENs, also called mitoTALENs. These mitoTALENs comprising a FokI nuclease are able to knock out certain mitochondrial genes, which has been demonstrated in broccoli (Xu et al. 2024), tobacco (Forner et al. 2023), potato (Nicolia et al. 2024), and rice (Kazama and Arimura 2023). Furthermore, TALE-based base editing systems using a DddA cytidine deaminase are being developed to induce base conversions in the mitochondrial as well as the chloroplast genome. Examples of such base editing systems are TALE-DdCBEs, which target C·G-to-T·A conversions (Zhang et al. 2024), TALE-DdABEs, which target A·T-to-G·C conversions (Zhang and Boch 2024), and TALE-linked deaminases targeting A·T-to-G·C conversions (Mok et al. 2022; Zhou et al. 2024). However, these organellar base editing systems lack any strand-specificity. To try to improve the strand-specificity of organellar base editing, Hu et al. (2023) developed the cyDENT organellar based editing system. This system comprises of a TALE-linked deaminase that is single-strand specific and reached a base editing strand specificity of 95% in mitochondrial genomic targets (Hu et al. 2023).

Crop varieties with genetically engineered organellar genomes have not been commercialized yet. Persisting challenges that may slow down the introduction of such crop varieties on the market include the abovementioned technical challenges but also public acceptance, regulatory challenges, and the required safety assessments (Zhang and Zhu 2024). However, field tests are already being performed for some varieties (Umemoto et al. 2023). Hence there is a possibility that the developed mitochondrial and chloroplast genome editing systems may soon be applied for improving commercial crop (Zhang and Zhu 2024).

### Advancements in random mutagenesis

Besides advancements in gene editing, new genetic technologies utilizing random mutagenesis have been in use for plant breeding purposes as well. These technologies comprise of particle mutation breeding (high- and low-energy) and space mutation breeding. Particle mutation breeding make use of ionizing particles with high linear energy transfer (LET). These high LET properties of ionizing particles cause them to be more mutagenic than conventional X-rays and gamma-rays (Huefner et al. 2014). This higher mutation frequency is caused by the type of DNA damage generated by particles with high LET: they induce a substantial amount of damage in only a small area within the DNA, also called “clustered DNA damage”, in the form of SSBs, DSBs and diverse DNA lesions (Jo and Kim 2019). These damaged clusters caused by high LET particles have proven to be hard to repair, leading to free DNA fragments, causing chromosome rearrangements and large deletions. Consequently, the variety of gene mutations and the new trait frequency during breeding are increased (Ma et al. 2021; Udage 2021).

Most commonly used particles for the purpose of mutation breeding are heavy ions, such as argon-, carbon-, iron-, lithium-, and nitrogen- ions, whilst protons, fast neutrons, electrons, and alpha particles are also used for breeding applications (Ma et al. 2021). Energy can be transferred to these particles in particle accelerators, in which the positively charged ions are given a high speed of 20–80% of the speed of light (Maurya et al. 2022). The energy levels of ionizing particles in so-called ion beams can range from high to medium or low. High-energy ions are defined as ions containing radiation doses of more than 200 kiloelectron volts (KeV), but can potentially contain more than thousands of megaelectron volts (MeV) (Ma et al. 2021). High-energy heavy-ion beams generally induce more large-scale DNA rearrangements such as chromosomal rearrangements and large deletions (Hirano et al. 2015) compared to conventional X-rays and gamma-rays, inducing more small mutations such as base substitutions and small insertions and deletions (Jo and Kim 2019; Li et al. 2019).

On the other hand, low-energy particle mutation breeding is performed with particles containing low amounts of ionizing radiation energy (10–200 keV). When containing low energy, heavy-ion particles have only short penetration depth (Yu and Anuntalabhochai 2012), causing a small part of the surface cells of the plant embryo to be exposed to radiation (Li et al. 2011). However, low-energy heavy-ions still have mutation inducing effects due to processes called long-distance or radiation-induced bystander effects (RIBEs). These RIBEs are responsible for phenotypic changes in non-irradiated cells, caused by cells that are damaged by irradiation and transfer signals to non-irradiated cells (Ma et al. 2021). The exact mechanism of RIBEs is still subject for debate, although it is expected that two main pathways for signalling between the irradiated cell and the non-irradiated cell are involved, namely the transmission of these so-called bystander signals via cell-to-cell contact and via the release of these signals into culture medium or plasma (Ma et al. 2021). Moreover, it is thought that irradiated cells also excrete low-molecular-weight factors, such as reactive oxygen species (ROS), cytokines, calcium ions, and small RNAs, that are able to induce bystander effects in non-irradiated cells (Merrifield and Kovalchuk 2013). Directly radiated cells can transmit bystander signals as a consequence of a large variety of radiation-induced DNA damage effects, such as induced mutations (caused by SSBs, DSBs, or base damage), altered gene expression, chromosomal instability, and sister chromatid exchanges (Szumiel 2003) (Desouky et al. 2015). While the type of mutations of directly low-LET particle-irradiated cells consist of mainly deletions, the type of mutations occurring in the non-irradiated bystander cells consist of mainly point mutations (Huo et al. 2001).

Another random mutagenesis technique that has been used to create a large amount of crop varieties since 1987 in especially China is space mutation breeding (Pei et al. 2019). Space mutation breeding is performed by bringing plant seeds onto a spaceship in low earth orbit and consequently exposing seeds to cosmic ray irradiation containing, among others, high-energy protons, heavy-ions, and high atomic number and energy (HZE) particles (Ma et al. 2021). Especially HZE particles are important for space mutation breeding, since these particles can invade the spacecraft cabin and produce mutation inducing secondary particles. The average dose rate of HZE particles is generally low, although their peak energy can reach 1,000 MeV with a LET of 100 keV/µm (Ma et al. 2021). Long-term exposure to HZE particles and other sources of radiation in the space environment may cause clustered DNA damage and DSBs. Because of the dense ionizing track and thus the relatively high mutagenic property of HZE particles, DNA damage caused by these particles is slowly repaired and difficult to repair correctly (Asaithamby and Chen 2011). Moreover, DNA mutations caused by cosmic ray irradiation in the space environment are not randomly distributed throughout the DNA. Mutations caused by space breeding are suggested to occur more frequently in polymorphic clusters in the DNA, which are regions in the DNA that are changed more easily by external factors (Li et al. 2007).

Apart from the developments in ionizing radiation sources used in radiation breeding, mutagenic effects of radiation may eventually become more predictable with the progression of sophisticated radiation tools and modern biotechnologies such as high-throughput gene sequencing (Ma et al. 2021). An example of such a high-throughput selection tool is called Targeted Induced Local Lesions in Genomes (TILLING). TILLING is a genetic screening method that enables more efficient and accurate selection of mutations in a population than phenotypic screening and allows identification of heterozygous recessive traits as well (Udage 2021). TILLING comprises of a combination of random mutagenesis through low doses of a chemical mutagen combined with a sensitive mutation detection instrument. A delicate balance needs to be struck between the mutation frequencies and the lethality of the dose of the chemical mutagen, such as ethylmethanesulfonate or nitrosomethylurea. This is done in order to create a population of plant materials with adequate numbers of mutants. Doses used might therefore be lower than for traditional forms of random mutagenesis. Radiation mutagenesis is generally not preferred due to additional effects, such as chromosome breakage, which are not useful (Ritchie and Nielsen 2006). This genomic sequence screening methodology was developed in the 1990s and first commercially used in 2000. With this methodology, the predominantly point mutations induced by the chemical mutagen are followed by high through-put mutational screening for discovering induced lesions (Roychowdhury and Tah 2013). TILLING can aid in the identification of new functions of genes, SNPs, and InDels in a gene of interest, and the biochemical function of a gene (Roychowdhury and Tah 2013).

### Null segregants from plants containing transgenic elements

Another type of plant that can pose challenges for the enforcement of GMO regulations are null segregants. Null segregants can be defined as "the non-transgenic progeny of a transgenic parental line” (Camacho et al. 2014). It is important to stress that null segregants by themselves are not a technique but rather a result of normal segregation that can occur also in conventional crossings. The advantage of using null segregants in combination with transgenic techniques is that transgenic traits can be used in the breeding process without these traits being present in the final plant product. In this section, we will describe various ways to accelerate breeding, to create hybrids, and to induce gene edits or epigenetic changes that encompass the use of null segregants.

#### Null segregants in accelerated breeding

Early flowering approaches encompass the introduction of a transgene that leads to more rapid development of flowers, thereby shortening generation times. This enables more crosses in a shorter timespan and quicker introduction of desired traits (*e.g.*, disease resistance). For example, by introducing and overexpressing the *BpMADS4* gene from silver birch in apple lines (Flachowsky et al. 2011), researchers were able to introduce the naturally occurring fire blight resistance gene from the ornamental apple cultivar in a commercial food apple cultivar “Pinova” within 7 years (Schlatholter et al. 2018). Moreover, this transgenic line could be used to introduce blue mould resistance from a wild apple into a commercial Gala cultivar (Luo et al. 2020). Overexpressing *Flowering Locus T1* from poplar in plums leads to an early flowering phenotype (Srinivasan et al. 2012). This enables introducing resistance to plum pox virus from one plum variety into another and breeding stoneless plums (Petri et al. 2018). The flowering locus T has also been introduced in other crops, such as citrus (Endo et al. 2020), cassava (Bull et al. 2017), eucalyptus (Klocko et al. 2016), pear (Tomes et al. 2023), and kiwifruit (Varkonyi-Gasic et al. 2019), although these are all in research settings. Early flowering approaches are particularly relevant for crops with relatively long generation times, whereby the first flowering normally occurs after a juvenile phase of several years (Tomes et al. 2023). These approaches are most effective when combined with marker assisted selection for efficient selection of desired traits (Endo et al. 2020; Flachowsky et al. 2011).

#### Null segregants for hybrid seed creation

Transgenic technologies may be used to help create hybrid seeds, whereby the hybrids are null-segregants. Hybrid varieties usually have higher yields and better resilience than non-hybrid varieties and are bred from two largely homozygous lines.

Seed production Technology (SPT) facilitates hybrid production through a system with a transgenic maintainer line, a male-sterile line, and a fertility restorer line (Wu et al. 2016). The transgenes from the maintainer line are absent from the hybrid seeds destined for agriculture. For maize, a few SPT variants have been developed (Fox et al. 2017; Qi et al. 2020; Wu et al. 2016; Zhang et al. 2018). The SPT system consists of a fertility restoring gene (often the wildtype variant of naturally occurring male-sterility alleles), a pollen-specific gene that prevents pollen formation (*e.g.*, alpha-amylase or a mitochondrial disruptor), and a seed-specific colouring gene for easy selection (Wan et al. 2019; Wu et al. 2016). These systems may be extended with other genes, such as in the “multi-control sterility” system in maize, which also contains a DNA adenine methylase to devitalize pollen and a herbicide-resistance gene for transgenic seed selection (Zhang et al. 2018). Maize hybrids developed with SPT are commercialized in the USA. Likewise, rice systems based on GM maintainer lines with null-segregant offspring have been developed for agricultural use. These systems are heralded as “third-generation hybrid rice technology” (Chang et al. 2016; Sheng et al. 2023; Song et al. 2021).

**Fig. 4:**
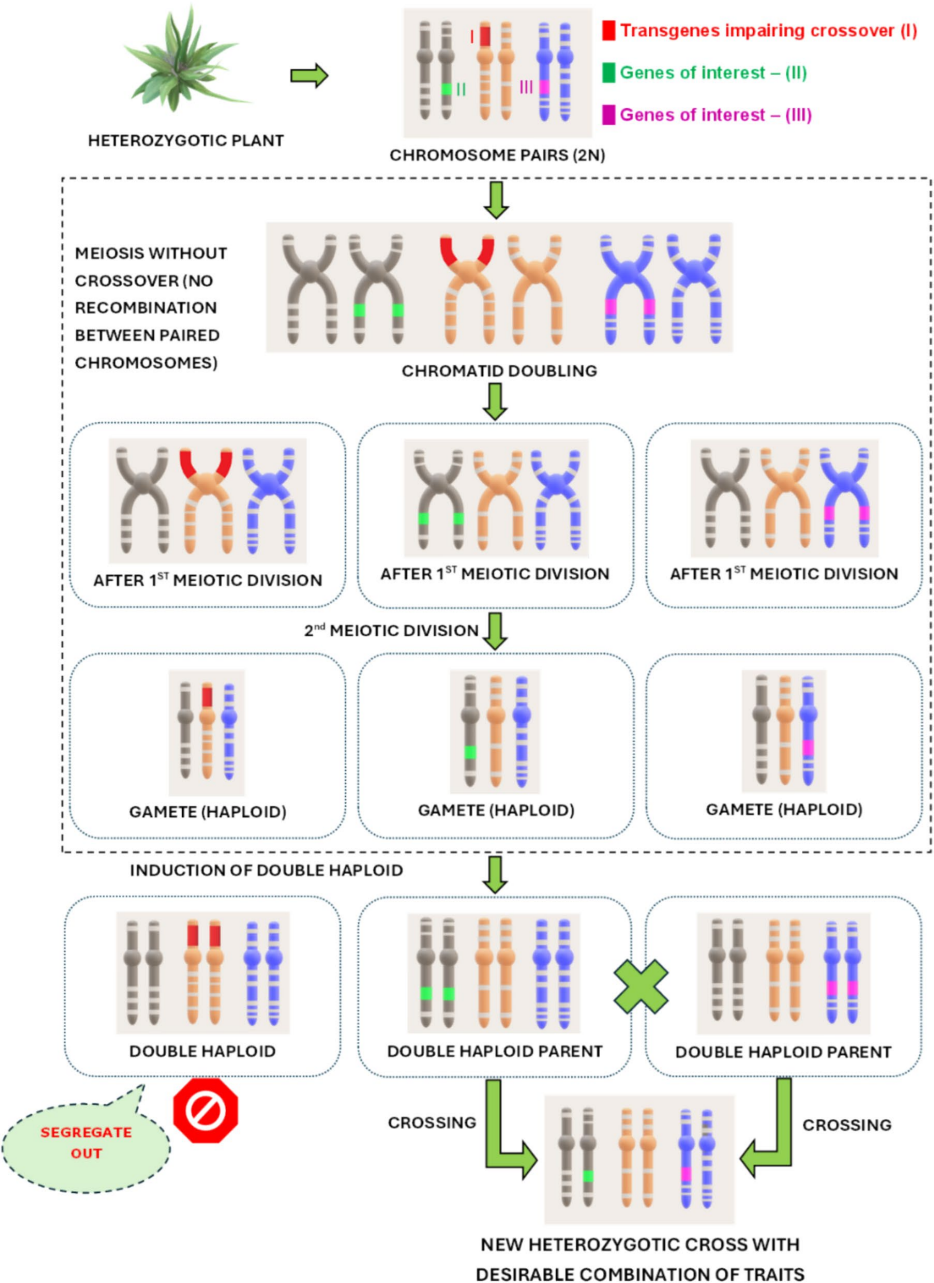
Principle of reverse breeding through the creation of double haploid parents derived from a heterozygotic plant. Transgenes are temporarily introduced to help avoid cross-over of chromosome segments during meiosis. Whereas crossover naturally occurs during meiotic division, it would interfere with the preservation of the genetic makeup of the initiator plant. In the final double haploids selected for further breeding, the transgenes (red) are lost through segregation, while the genes of interest (green, lilac) are still maintained

With reverse breeding, two homozygous parental lines can be obtained from an elite hybrid plant through meiotic suppression. This is achieved through a combination of RNA interference via transgenesis and doubled haploid technology applied in the elite hybrid (Lusser et al. 2011). Using these technologies, two plants with a complementary genetic composition that do not contain the transgene can be created, selected, and subsequently used for the production of seeds (Fig. 4). At the time of this writing, it is not clear if any products of reverse breeding have been submitted for market approval or have been placed on the market in non-EU countries.

Doubled haploids provide an alternative for inbred breeding, such as the haploid-Inducer-Mediated Genome Editing (IMGE) system (Wang et al. 2019) and the Syngenta/Northrup King’s Haploid induction-edit (HI-Edit) system (Kelliher et al. 2019). These systems combine haploid induction with gene editing during fertilization.

#### Null segregants in gene editing

The gene editing machinery may be delivered to a plant via introducing DNA cassettes harboring CRISPR/Cas9 components, TALEN or ZFN genome editing machineries (Wada et al. 2020). The result is a transgenic plant in which desired DNA changes can be induced. In subsequent breeding steps, progeny of the transgenic plant is selected, and this progeny has the desired edit but lacks the transgenic traits for the gene editing machinery. Marketed examples of such null segregants with gene edits are the Calyno™ high-oleic acid soybeans with inactivated fatty acid desaturase 2 genes (Haun et al. 2014; Kim 2021) and Waxy CRISPR Corn from Corteva, wherein the Waxy gene is deleted. However, the gene editing machinery can also be delivered to the plants in ways that do not rely on segregation and the selection of null segregants. For example when direct RNP delivery is used, no transgenic DNA encoding gene editing components are introduced, circumventing the need to select null-segregants. As “DNA-free” RNP-mediated genome editing is efficient (Wada et al. 2020), it is likely to become a method of choice for gene editing crops in the future. In such a case, the issue of null segregants does not apply.

#### Null segregants and epigenome editing

Transgenic parental plants can also be used to induce epigenetic changes in the null-segregant offspring. Upon introducing a construct with an inverted repeat of the target gene, the plants’ RNA-directed DNA methylation process results in methylation of that target gene. Next, a null-segregant containing the desired epigenetic edit but not the construct can be selected (van de Wiel et al. 2016). This technology has been used to silence *Msh1* expression in soy (USDA APHIS 2017). Nonetheless, at the time of this writing, no market applications of RNA-directed DNA methylation have been identified (Parisi and Rodriguez-Cerezo 2021).

## Regulation of crop trait modifications using innovative breeding approaches

The legislation of crops obtained with modern biotechnology may vary from one nation to another. In addition, different agencies within the same country overseeing different aspects of the same innovative crop may have divergent approaches. In case authorization is needed, a pre-market risk assessment is commonly part of the procedure that a crop developer has to go through. For this, it will have to provide a dossier with safety data to the authorities. This adds to the requirements for variety testing, plant breeders’ rights, etc. that already apply to any new variety of crop species with variety protection. The same genetic modification may actually be bred into multiple varieties with their genetic and phenotypic characteristics adapted and optimized towards the local circumstances.

Some but not all nations have adopted the definition of “living modified organisms” of the Cartagena Protocol under the International Convention on Biological Diversity as basis for their national legislation on modern biotechnology. The Protocol covers the international movement, biosafety, and information exchange about living modified organisms. This protocol (Article 3) defines them as carrying “new combinations of genetic material” obtained through “modern biotechnology”, hence including both product-and process-based triggers, respectively. The concept of “modern biotechnology” includes in-vitro techniques such as recombinant DNA or direct injection of genetic material, as well as fusion of cells from species that are naturally incompatible (CBD 2003).

The legislations regarding biotechnology-derived crops vary considerably between various nations across the globe with regard to coverage of gene-edited crops  (Table 2). To give an elaborate overview of these differences, we describe the regulations for biotechnology derived crops in place in the following nations or regions: Argentina, Japan, the EU, the UK, the USA, Canada, and Australia and New Zealand. These are each major importing and exporting nations or regions of agricultural commodities with elaborate national legislations regarding biotechnology derived crops. Also, these regions have a high accessibility of information on legislations in English from their own governments.

**Table 2:** Overview of the coverage of crops with small gene edits by modern biotechnology regulations in selected countries and regions

Country/region	Small gene edits regulated as GMO?	Responsible authority	Regulatory status and additional information
Argentina	No	CONABIA	Not considered a living modified organism (according to the same definition as in the CBD)
Australia and New Zealand	No	OGTR, FSANZ, MPI	SDN-1 defined as non-GMO (NZL pending)
Canada	No	Health Canada, Canadian Food Inspection Agency	Transgenic products considered novel, which is not necessarily the case for gene-edited ones depending on the trait and characteristics of the crop and food
European Union	Yes	European Commission	Status may change to non-regulated as GMO but as a separate category under amended legislation for new genomic techniques, which is still in preparation
Japan	No	Ministry of Agriculture, Fisheries and Food; Ministry of Health Labor, and Welfare	SDN-1 defined as non GMO
United Kingdom (England)	No	Secretary of State for Environment, Food, and Rural Affairs	Not regulated as GMO but as the separate category of “precision-bred” crops if comparable to traditional breeding outcomes
United States of America	Yes/No	USDA, EPA, FDA	USDA would still consider it “genetic engineering” whilst the deciding criterion then is the potential to become a plant pest or weed; EPA and FDA focus on the risk characteristics of the product as trigger for regulatory action

### Argentina

In 2015, Argentina became the first country to issue legislation that specifically focused on gene-editing and other new breeding techniques in plants (Whelan and Lema 2015). A key question is whether or not Argentina defines a new crop as a GMO, according to the Cartagena Protocol’s clause of “new combinations of genetic material”. This implies that the host genome has been changed by the insertion of a DNA sequence construct in a stable and cohesive way. Under this definition, certain gene-edited crops would not be considered GMOs based on the nature of the introduced modifications, such as small mutations. After a review, an update and simplification of this regulation took place in 2021. This also included a procedure for applicants to consult with the authorities at an early stage of product development on the regulatory status (GMO or not) of their product. This would then be followed by another consultation at a later stage to verify whether assumptions made earlier were correct (Goberna et al. 2022).

### Japan

Notably, Japan has implemented a similar procedure as Argentina in which developers of gene-edited plants can do a “pre-submission consultation” of a committee advising the Ministry of Health, Labour, and Welfare at an early stage of product development. An important detail for the determination of “gene-edited” status with limited data required is whether “foreign” DNA is still present within the edited host plant. Notably, also “self-cloned” plants with insertions containing DNA from only the same or crossable species would be eligible, implying that cisgenic plants would also be included in the bracket of eligible crops (Kondo and Taguchi 2022).

### EU

Some other countries follow a process-based approach. Even though they may exempt organisms that have been obtained with modification methods that have already proven to be safe, they may not include gene editing and other new breeding techniques in this bracket. This is the case in EU legislation, for example, in which the definition of GMOs is broad. EU legislation defines GMOs as having genetic material that “has been altered in a way that does not occur naturally by mating and/or natural recombination” (EU 2001a). Notably, this unnaturalness can apply to both the method used to change the genetic material and to the resulting change in the genetic material itself. Crops derived from “traditional” forms of in vivo random mutagenesis involving the exposure of plant parts to radiation or mutagenic chemicals also qualify as “genetically modified” under this definition. Space breeding and particle-beam mutagenesis can be regarded new variants of radiation-based random mutagenesis. Yet EU legislation explicitly exempts plants obtained with these techniques from the scope of legislation on GMOs. This is because there is a history of application of these techniques among plant breeders (CJEU 2018).

By contrast, if developers use modern, targeted molecular techniques such as CRISPR-Cas, the resulting plants would not fall under this exemption regardless of whether the changes obtained could be the same as those obtained through random mutagenesis. The reason for this is that these techniques are novel as they were largely introduced after the adoption of the GMO legislation in 2001 (CJEU 2018).

At the moment of writing, there is still debate around a proposal from the European Commission for amendment of EU legislation on the so-called new genomic techniques (NGTs) applied in plant breeding. These include both targeted mutagenesis and cisgenesis. The latter concept entails modification of crops with genes with unchanged sequence from the breeder’s gene pool. This pool includes other varieties of the same crops and wild relatives that can be crossed with the host crop. “Fast-track” procedures would particularly apply to the proposed “category 1” of “NGT plants”, that are considered to be equivalent to conventional plants. The criteria for equivalence include DNA deletions or inversions of any size, substitutions or insertions of 20 nucleotides or less, or the insertion of non-interrupted DNA segments that also occur in the breeders’ gene pool. In addition, this also includes any other targeted modification of the crop DNA provided that the changes caused already occur in the breeders’ gene pool (European Commission 2023b) (European Commission 2023a).

### UK

In 2023, the UK enacted the Precision Breeding Act, which applies to plants and animals alike. It defines “precision breeding” as the use of modern biotechnology to introduce stable genetic changes which could in theory also be introduced via “traditional processes” such as conventional crossing, random mutagenesis, rootstock grafting, polyploidy introduction, embryo rescue, or somatic cell fusion of cells from species that can also be crossed naturally. Under the act, a person can sell or grow a precision-bred organism in England after having received confirmation from the Secretary of State of the Department for Environment, Food, and Rural Affairs (DEFRA). For this, the applicant has to submit required information with the application. An advisory group will then assess these data and advise the Secretary before replying to the application (UK 2023). At the time of writing, secondary legislation and technical guidance are being developed with the aim of faciliting the confirmation procedure (Stockdale 2024).

### USA

Under the Plant Protection Act, the USDA oversees the interstate transport and movement, field testing, and cultivation of “genetically engineered” crops under the Plant Protection Act. Such crops by default have to be risk-assessed for the possible risk of becoming pests or weeds. Under the “Am I Regulated?” procedure, developers can enquire with USDA APHIS whether their product is regulated and would therefore require authorization. In case the GM plant poses no risk of becoming a plant pest, contains no foreign plant pest DNA, and/or is incapable of self-propagation, it may be considered non-regulated and therefore not subject to authorization (USA 2024; USDA 2024a, 2024b; USDA APHIS 2019).

Besides the regulatory provision of the USDA, those of the Environmental Protection Agency (EPA) and the Food and Drug Administrations (FDA) may apply as well. The EPA oversees the uses of “plant-incorporated protectants (PIPs)”, such as newly expressed insecticidal proteins within transgenic crops. It recently introduced a new rule exempting PIPs that are identical to those from crossable plant species. Also exempt are proteins from the host crop that carry mutations or loss-of-function that conventional breeding could also have caused (US EPA 2023).

The US FDA, which oversees food safety, follows a product- or actually risk-based approach. GM-crop-derived new food or pet food components are therefore not categorically included or excluded from the requirement to undergo authorization as a “food additive” prior to commercialization. A food additive in this case is a substance that is added to food (intended to become a component of food) and not inherently part of it. If already present, the substance is still a food additive if its levels have risen by human intervention. These would include, for example, new proteins introduced by genetic modification into a crop. Exempt from this definition are substances that are “generally recognized as safe” (GRAS). For food additives, the next question is then whether it may be injurious to health. This could be the case when the level of a natural plant toxicant within the host plant has been raised by the genetic modification. The latter scenario could also equally well apply to a conventionally bred plant, though. Developers may determine by themselves that their product is GRAS. Yet the FDA can still take action against the distribution of the implicated food if it finds the product to be non-GRAS instead. FDA encourages developers to voluntarily seek advice about the regulatory status of their product, which is done by many biotechnology developers (Kok et al. 2019; US FDA 1992). Recently, the FDA published a statement for developers of genome-edited foods. It referred to the common occurrence of mutations and unintended effects as well as crop breeding practices singling out off-types. Yet it did not preclude that the application of new techniques could lead to effects that would still require a risk assessment. Examples of such effects could be changes in proteins that would modify their allergenicity, impacts on nutritional value, or the introduction of new metabolites of concern or increased levels thereof (US FDA 2024).

### Canada

Canadian legislation focuses on the novelty of the product. This applies to both the so-called “plants with novel traits” and “novel foods” which come under the regulatory scrutiny of the Canadian Feed Inspection Agency and Health Canada, respectively. Vice versa, besides some GM varieties of crops, various plants with novel traits that underwent pre-market assessment include non-transgenic herbicide-tolerant crop varieties that had been obtained via somaclonal variation. Both regulatory agencies recently reviewed the possible impact of gene-editing, particularly in terms of the intended and unintended effects and how these compared to those obtained through conventional breeding and recombinant DNA techniques, as well as the safety net that breeding practices form against unintended undesirable effects. They concluded that no changes to the current “product-based” regulatory regime was necessary, that is, no separate mandatory pre-market requirement for gene-edited crops would have to be imposed (CFIA 2023; Health Canada 2024).

### Australia and New Zealand

Australia and New Zealand’s joint Food Standards Code discerns “foods producing gene technology”. It defines the latter technology as the use of recombinant DNA techniques to genetically modify organisms or cells. Food Standards Australia New Zealand recognizes, though, that some of the changes in foods brought about by “new breeding techniques” may be equivalent to those obtained with conventional breeding. To provide clarity, the Code was amended in September 2025, excluding such cases from the definition of “gene technology”. This also holds true for foods derived from null segregants as well as refined food ingredients that do not contain any novel protein or DNA anymore. A genome-edited, food-producing organism is also exempted from the definition as long as the modification does not entail the introduction of novel DNA. For this reason, also cisgenesis-derived foods are exempt. The authority cautions, though, that even though certain food-producing organisms obtained with new breeding techniques may be exempt from the requirements for GM foods, they still may require regulatory approval as “novel foods” if their traits have been altered substantially. This is no different from novel foods from conventional sources (FSANZ 2025).

Environmental releases and confined uses of GM crops in Australia require a licence from the Gene Technology Regulator. For advice, the Regulator, in turn, can consult two expert advisory committees, one being technical and the other focused on ethical and social aspects. The regulatory framework is the Australian Gene Technology Regulatory Scheme. It contains a set legislative pieces that both state and territory authorities have to enforce. Also here, reference is made to “gene technology”. In 2021, the Office of the Gene Technology Regulator (OGTR) clarified that site-directed nuclease-1 (SDN-1)-derived organisms are not GMOs. Besides SDN-1 derived organisms, null segregants are no GMOs either (OGTR 2021).

In New Zealand, the regulatory system has been relatively prohibitive towards the use of GMOs. Yet the New Zealand government is currently transforming its regulatory system for GMOs. For instance, it will install a Gene Technology Regulator based on the Australian example. The new framework is to start in 2025. Under the new framework based on Australia’s Gene Technology Act 2000, gene-edited organisms are likely to become exempt from regulation (NZ MBIE 2024).

## Discussion

Our review covers a wide range of genetic crop improvements that are being explored by academic and corporate researchers and breeders worldwide and are in some cases also brought to the market place. Notwithstanding this diversity, we can infer a number of general observations across these developments.

For instance, it is striking that also in the field of “conventional” random mutagenesis, new methods and refinements are being developed and successfully implemented. Examples include the use of ion particle beams and “space breeding” for radiation mutagenesis as well as TILLING as a refined chemical mutagenesis method. Closer scrutiny shows that although the frequency and spatial distribution of the induced mutations may differ, they are still of the same nature as in older methods. This also holds true for various recent molecular methods, such as nucleases used for gene editing and base editing as well as mobile genetic elements. Yet this also points at a potential inequal treatment of the random mutagenesis techniques as compared to the more precise, “surgical” DNA enzymes such as CRISPR Cas and TALENs. Moreover, techniques of random mutagenesis are considered to have a history of safe application over decades. Yet this review shows that this field is not that static but more dynamic.

An important consideration in the safety assessment of gene-edited and other GM crops is also the potential unintended effects of the modification. In gene editing, particularly the potential for “off-target” mutations at sites showing homology to the target site is one of the key considerations. If such unintended effects were to occur, the procedures followed by plant breeders would act as a safety net. To obtain homozygous breeding lines, breeders do many rounds of backcrossing and discard plants showing undesirable effects on crop appearance or performance. This is different from applications in, say, the animal or human domain, in which off-target effects in the first generation of cells are undesirable. This is because of their immediate impacts on the health and welfare of the host and the inability to do extensive forward and backward-crossings. This has to be taken into account when trying to extrapolate literature on off-target effects of gene editing tools applied in non-plant targets to plants. Moreover, the regulatory pre-market scrutiny of such crops could accommodate methods to characterize any persisting off-target modfications, as an additional safety net (Lema 2021).

The review of gene editing regulations in various nations across the globe shows that authorities have already been making regulatory arrangements for the advent of gene-edited crops and other new technologies to the market. International harmonization of regulatory food safety assessment of recombinant DNA crops has already been achieved via the Codex Alimentarius guidelines published in 2003. In fact, the Codex Alimentarius guidelines could potentially also be applicable to the food safety assessment of gene editing-derived products. Whether specific elements of the safety assessment recommended by these guidelines apply needs to be determined on a case-by-case basis (FAO 2023).

Some gene editing methods involve the transient introduction of the editing machinery into cells or organisms, resulting in null-segregants. These null-segregants have been freed of transitionally present constructs, and questions can be raised on their regulatory status. Some legislations, such as in the US, Japan, or Australia explicitly exclude them from their regulations, whereas their regulatory status is unclear for some other regions, such as the EU. This also raises questions about enforcement of these regulations, as it does for gene-edited crops per se, given that these are indistinguishable from non-modified counterparts of the same crop species.

Another general observation is that the outcomes of many studies would not hint at potential food safety issues. Yet only a few publications in this domain explicitly pay attention to food safety-related issues, such as the potential for off-target or unintended effects. A few historical examples exist where the plant breeding process led to unsafe varieties. For example, in the 1990s, plant breeders aimed to develop celery with improved nutrient content. However, some of the varieties contained enhanced levels of furanocoumarins, a potential toxicological risk for consumers (Bruznican et al. 2020). Furthermore, in the 1960s and 1970s, novel starchy potato breeds, Lenape varieties, were found to contain toxic levels of glycoalkaloids (*e.g.*, solanine) (Sinden and Webb 1974). Cases such as these have made plant breeders and regulators aware of the potential presence of toxicants in food crops and have led to the adoption of variety assessment protocols. More pro-active practices and a safety culture implemented amongst developers can aid in the safe development of new crop innovations through genetic technologies, for example through “Safe-by-Design” approaches (Groenen et al. 2025; van der Berg et al. 2020). Particularly if food safety-based considerations can steer the product development process in a risk-proportionate manner, this may help prevent failures for safety reasons further down the line. Also, taking such considerations into account might aid in ensuring food safety of novel products with which legislation has not managed to keep pace.

Whereas this review focuses on safety assurance for new genetic technologies applied to food crops, this is not the sole factor that developers and decision makers need to address so as to acquire a “licence to operate” from the general public. This becomes evident, for example, from the recent discussions around the proposed legislative amendments for crops obtained with NGTs within the European Union. Here, several points of contention and debate are involved. These include the issues of patentability and its potential perturbation of plant breeders' rights and labeling of gene-edited foods at the retail stage so as to maintain freedom of consumers’ choice (European Parliament 2025).

## Concluding remarks

In this perspectives review, we describe advancements in a large diversity of genetic crop improvement techniques. There are continuous developments in newer but also in more conventional fields, which may give rise to regulatory challenges. No method-specific food safety concerns arise based on the type of mutations induced with these newly developed techniques. However, since potential unintended effects of these technologies are as of yet underexplored, more pro-active safety practices would be of added value to developers of genetic crop improvement techniques.

## Data Availability

No datasets were generated or analysed during the current study.
